# *Alms1* KO Rat: A New Model of Cardiometabolic Syndrome With Spontaneous Hypertension

**DOI:** 10.1111/apha.70174

**Published:** 2026-02-15

**Authors:** Ankita B. Jaykumar, Sumit R. Monu, Jiang Xu, Mariela Mendez, Xiao‐Ping Yang, Nour‐Eddine Rhaleb, Pablo A. Ortiz

**Affiliations:** ^1^ Division of Hypertension and Vascular Research Henry Ford Hospital Detroit Michigan USA; ^2^ Department of Physiology Wayne State School of Medicine Detroit Michigan USA

**Keywords:** Alstrom syndrome, cardiometabolic syndrome, endothelial function, hypertension

## Abstract

Alström syndrome 1 (ALMS1) is a protein linked to Alström syndrome, a rare genetic disorder characterized by obesity, insulin resistance, hyperinsulinemia, and hypertension. Genetic studies have further associated *Alms1* with hypertension in human populations. However, the precise mechanisms by which ALMS1 regulates metabolic and cardiovascular function remain unclear.

**Aim:**

In this study, we investigate metabolic and cardiovascular functions regulated by ALMS1.

**Methods:**

To investigate this, we developed and characterized an *Alms1* knockout (KO) rat model, which spontaneously develops metabolic syndrome and hypertension.

**Results:**

Our findings reveal that *Alms1* KO rats exhibit age‐dependent metabolic dysfunction, with hypertension and increased body weight becoming evident by 10–12 weeks of age. Obesity, hyperinsulinemia, and vascular dysfunction emerge later, at 14–16 weeks, suggesting progressive metabolic deterioration. Notably, *Alms1* KO rats develop hyperleptinemia as early as 7 weeks, prior to the onset of obesity, implicating ALMS1 in early leptin regulation and metabolic signaling. Moreover, female *Alms1* KO rats develop severe metabolic syndrome with hypertension, like males, demonstrating a lack of the typical female cardiovascular protection. Echocardiographic analysis shows progressive cardiac dysfunction, including left ventricular (LV) dilation, increased wall thickness, and impaired contractility. Despite these structural changes, the LV mass/BW ratio remains unchanged, suggesting a shift toward maladaptive eccentric remodeling rather than hypertrophy.

**Conclusion:**

Collectively, these findings establish the *Alms1* KO rat as a robust preclinical model of metabolic syndrome. This model closely mimics human disease and provides a powerful tool for studying the mechanisms of metabolic and cardiovascular dysfunction as well as for testing potential therapeutic interventions.

## 1 Introduction

Metabolic syndrome is a clustering of cardiovascular risk factors associated with hypertension, insulin resistance, glucose intolerance, obesity, hypertriglyceridemia, hypercholesteremia, and low levels of high‐density lipoprotein [1, 2]. Pre‐menopausal women are relatively protected from metabolic syndrome compared to men. In contrast, postmenopausal women have a higher prevalence of metabolic dysfunction, with the age‐related loss of female sex hormones [1, 2, 3]. Several genetic loci and genes have been discovered that are individually associated with one or a few of the above‐mentioned phenotypes of metabolic syndrome [1, 2, 3]. To our knowledge, there is no single gene that, when deleted in animals, causes most phenotypes associated with MetS, including hypertension.

Alström syndrome is a rare, autosomal recessive genetic disorder linked to deleterious mutations in the *Alms1* gene, which causes multiple phenotypes that are risk factors for metabolic syndrome in addition to cardiovascular disease and chronic kidney disease. In Alström syndrome patients, renal function deteriorates progressively with age, and end‐stage renal disease is a common cause of death [4, 5, 6, 7]. Hypertension is a common risk factor for chronic kidney disease (CKD) [8]. SNPs in the *Alms1* gene are linked to decreased renal function (CKD gen) and were also linked to hypertension and increased pulse pressure status in a multipoint linkage analysis in 7 primary sibling samples of African American, Caucasian, and Mexican populations [9]. In addition, Alström syndrome is associated with a distinct cardiac phenotype, with over 40% of infants developing a transient but severe cardiomyopathy early in life and approximately 20% of patients exhibiting a later‐onset cardiomyopathy characterized by reduced left ventricular ejection fraction, impaired global longitudinal strain, and myocardial fibrosis [10]. This suggests that *Alms1* is a gene with deep penetrance on a wide cluster of metabolic phenotypes and could be an essential player in maintaining normal metabolic and cardiovascular health.

We previously showed that ALMS1 is involved in blood pressure regulation and control of renal function by promoting endocytosis of the Na^+^/K^+^/2Cl^−^ cotransporter NKCC2 in the kidney [11]. Another study showed that *Alms1* mutant mice displayed altered intracellular localization of glucose transporter‐4 (GLUT4) and decreased insulin‐stimulated trafficking of GLUT4 to the plasma membrane in adipocytes [12], suggesting a mechanism for the development of insulin resistance due to *Alms1* deletion. In addition, *Alms1* mutant mice develop early onset obesity and insulin resistance, but parameters for cardiac function, blood pressure, or plasma leptin were not measured in either these *Alms1* mutant mice or in other *Alms1* animal models [12, 13, 14, 15, 16]. Therefore, it is unclear whether *Alms1* gene deletion causes the whole range of symptoms of metabolic syndrome including obesity, insulin resistance, hyperleptinemia, and altered lipid profile, as well as altered cardiac and endothelial function in addition to hypertension.

In this study, we characterized an *Alms1* knockout rat generated on the genetic background of the Dahl Salt‐sensitive rat. Our findings demonstrate that this model develops age‐dependent, sex‐independent obesity, hypertension, hyperleptinemia, hyperlipidemia, cardiac remodeling and dysfunction, and vascular impairment. Notably, this *Alms1* knockout rat exhibits all hallmarks of metabolic syndrome spontaneously, without any dietary intervention, making it a valuable tool for studying disease mechanisms and testing novel therapies for metabolic syndrome. Additionally, our data highlight the pleiotropic role of ALMS1 in metabolism and vascular function, reinforcing its significance in cardiometabolic health.

## 2 Results

### 2.1 *Young Alms1* KO Rats (6–7 Weeks Old) Exhibit Few Metabolic Abnormalities

**FIGURE 1:**
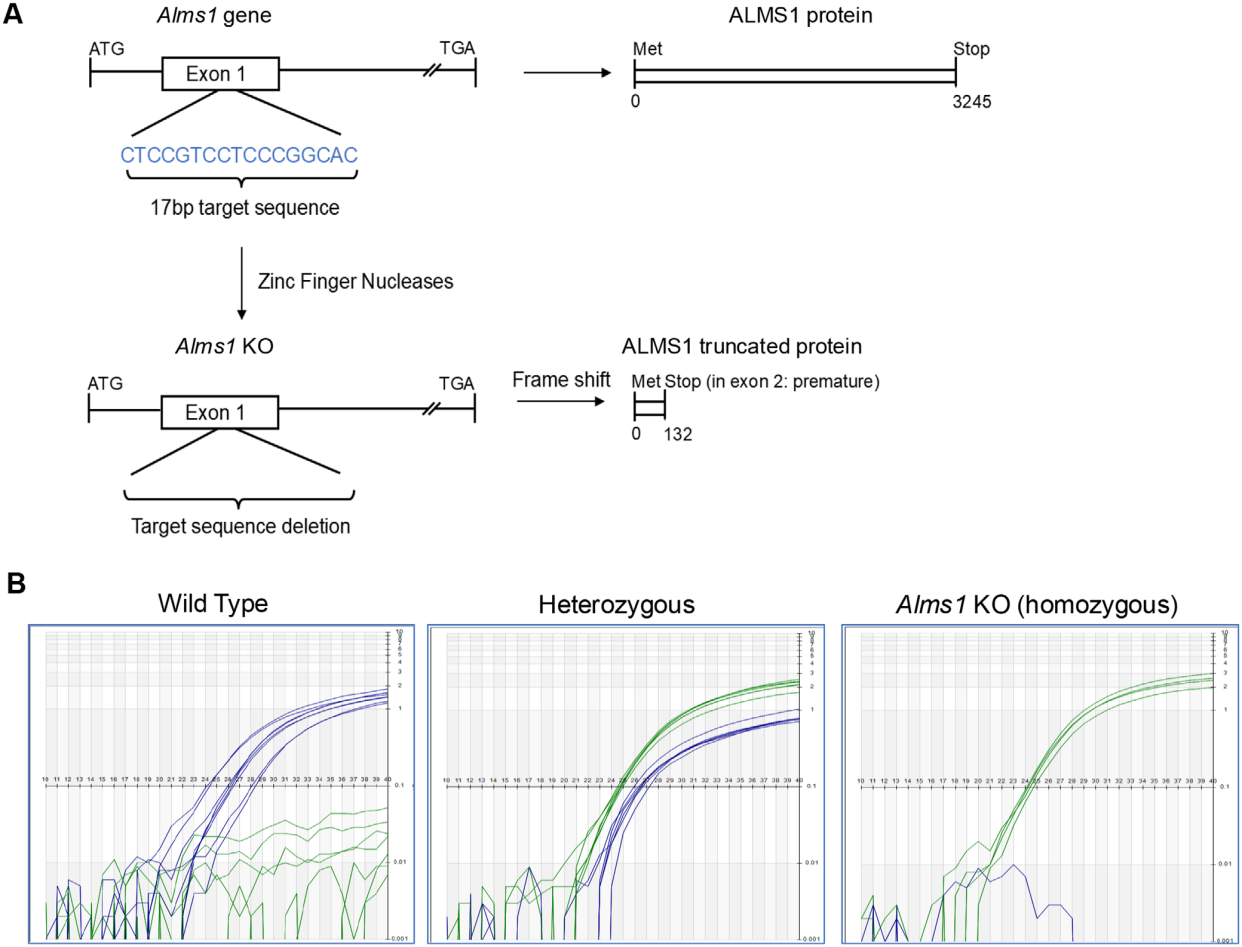
Generation of *Alms1* KO rats. (A) Zinc finger nucleases target 17 base pairs in exon 1 of the *Alms1* gene leading to a frameshift and causing a pre‐mature stop codon in exon 2 (figure adapted from [11]). (B) Representative Real‐Time PCR genotyping data for WT, *Alms1* KO homozygous and heterozygous rats.

Previous reports have shown that *Alms1* mutant mice are characterized by age‐dependent obesity, metabolic syndrome and kidney damage [13]. *Alms1* KO rats were generated as outlined in the schematic diagram (Figure 1A) and genotyped to confirm the knockout (Figure 1B). At age 6–7 weeks, there was significant variability in body weights in *Alms1* KO rats, with a tendency toward higher weight that did not reach statistical significance in Table 1 but was higher in other groups (Table 2). *Alms1* KO rats from both sexes displayed a higher trend in body weights. We found no difference in random plasma insulin or blood glucose levels between the strains or the sexes. Importantly, plasma leptin was extremely high at 6–7 weeks of age in male KO rats (*Alms1* KO: 3.53 ± 0.3 vs. WT: 0.17 ± 0.02 ng/mL) as well as in female KO rats (*Alms1* KO: 2.24 ± 0.52 vs. WT: 0.076 ± 0.024 ng/mL) (Table 1). These data indicate that at 6–7 weeks of age, *Alms1* KO rats may not have yet developed significant metabolic syndrome. However, the elevated plasma leptin levels observed in young *Alms1* KO rats preceded the onset of metabolic dysfunction, suggesting that ALMS1 may play a previously unrecognized role in leptin biology including its function, expression, and/or release.

**TABLE 1:** Six to seven‐week old *Alms1* KO rats have a normal metabolic profile.

Metabolic parameters	WT‐male	*Alms1* KO‐male	WT‐female	*Alms1* KO‐female
Body weight (g)	202 ± 7	215 ± 6	150 ± 5	165 ± 7
Plasma leptin (ng/mL)	2.0 ± 0.4	3.5 ± 0.3*	0.076 ± 0.024	2.24 ± 0.52*
Plasma insulin (ng/mL)	2.0 ± 0.4	3.0 ± 1.0	1.9 ± 0.3	3.4 ± 0.7
Blood glucose (mg/dL)	98 ± 5	116 ± 9	93 ± 5	98 ± 4

*Note:* Blood glucose, plasma insulin, and body weight in WT and *Alms1* KO rats measured in 6–7‐week‐old were similar except for the plasma leptin which was elevated in the *Alms1* KO rats. All values represent mean ± SEM and statistical analysis was performed with two‐tailed Student's *t*‐test. Male WT *n* = 9; male *Alms1* KO *n* = 6; Female WT *n* = 8; Female *Alms1* KO *n* = 7; *p < 0.05 versus WT.

**TABLE 2:** Echocardiography findings in 6–7‐week and 14–16‐week‐old *Alms1* KO rats.

	Males		Females		Males		Females	
	6‐week‐old				14–16‐week‐old			
	WT	*Alms1* KO	WT	*Alms1* KO	WT	*Alms1* KO	WT	*Alms1* KO
*N* of rats	6	6	6	7	9	7	9	7
BW (g)	170 ± 2	210 ± 13*	136 ± 2	149 ± 2*	374 ± 7	441 ± 23**	228 ± 7	350 ± 17****
LV mass/BW	43.8 ± 3.3	41.5 ± 4.8	36.1 ± 1.2	34.8 ± 1.9	26.7 ± 0.6	25.1 ± 1.2	28.1 ± 0.9	28.2 ± 1.1
HR (bpm)	378 ± 6	388 ± 6	391 ± 11	406 ± 10	349 ± 5	365 ± 15	365 ± 9	360 ± 10
IVSTs (mm)	2.5 ± 0.05	2.51 ± 0.12	2.32 ± 0.07	2.29 ± 0.10	2.97 ± 0.08	2.76 ± 0.1	2.58 ± 0.06	2.82 ± 0.11
IVSTd (mm)	1.48 ± 0.03	1.59 ± 0.05	1.41 ± 0.04	1.42 ± 0.06	1.77 ± 0.02	1.79 ± 0.04	1.61 ± 0.02	1.88 ± 0.05***
PWTs (mm)	2.65 ± 0.08	2.45 ± 0.09	2.66 ± 0.05	2.30 ± 0.1	3.01 ± 0.05	2.91 ± 0.03	2.80 ± 0.09	2.76 ± 0.1
PWTd (mm)	1.76 ± 0.06	1.52 ± 0.04	1.68 ± 0.06	1.41 ± 0.05*	1.81 ± 0.06	1.9 ± 0.05	1.64 ± 0.05	1.87 ± 0.03**
LVDs (mm)	2.85 ± 0.11	3.17 ± 0.09*	2.42 ± 0.05	3.04 ± 0.11*	3.71 ± 0.09	4.66 ± 0.23***	2.71 ± 0.10	4.11 ± 0.18****
LVDd (mm)	6.10 ± 0.25	6.39 ± 0.14	5.47 ± 0.09	6.14 ± 0.13*	7.53 ± 0.14	7.78 ± 0.22	6.19 ± 0.13	7.15 ± 0.21**
LVAs (mm^2^)	7.6 ± 0.8	10.7 ± 1.1	6.1 ± 0.4	8.7 ± 0.9	14.4 ± 0.7	24.4 ± 2.3*	8.2 ± 0.4	20.3 ± 0.26*
LVAd (mm^2^)	23.9 ± 1.3	28.8 ± 0.9	20.2 ± 1.2	22.6 ± 0.8	39.5 ± 1.5	43.5 ± 2.9	26.3 ± 1.7	33.2 ± 2.8
SF (%)	53.1 ± 1.4	50.3 ± 1.1	55.7 ± 1.2	50.5 ± 1.5*	50.8 ± 0.9	40.2 ± 2.6***	56.2 ± 1.0	42.5 ± 2.1****
SV (μL)	251 ± 27	303.3 ± 22.4	232 ± 21	228 ± 27	376 ± 31	367.2 ± 27.5	284.3 ± 20.7	285.8 ± 31.6
CO (mL/min)	95.1 ± 10.3	117.6 ± 8.5	91.5 ± 10.1	91.6 ± 9.8	130.6 ± 9.7	132.4 ± 6.9	103.9 ± 8.1	102.7 ± 11.7
CI (mL/min/10 g)	56.2 ± 6.5	57.6 ± 6.3	67.5 ± 7.7	61.5 ± 6.9	34.7 ± 2.2	30.3 ± 1.6	45.9 ± 3.8	30.1 ± 4.3*
E/A ratio	1.49 ± 0.09	1.28 ± 0.04	1.43 ± 0.07	1.26 ± 0.09	1.18 ± 0.07	1.32 ± 0.34	1.26 ± 0.09	1.23 ± 0.11
E_DT_ (ms)	24 ± 1	35 ± 1	27 ± 3	30 ± 1	29 ± 1	37 ± 4*	30 ± 1	37 ± 3**

*Note:* Echocardiography findings in male and female WT and *Alms1* KO rats measured in 6–7‐weeks of age. *p < 0.05, **p < 0.01, ***p < 0.001, ****p < 0.0001 versus WT.

Abbreviations: BW, body weight (g); CI, cardiac output/10 g; CO, cardiac output; E/A ratio, ratio between E/A waves; E_DT_: deceleration time of the early mitral filling wave (ms, milliseconds).; HR, heart rate (beats/min); IVSTs/d, interventricular septum thickness in systole and diastole; LVAs/d, left ventricular area in systole and diastole; LVDs/d, left ventricular dimension in systole and diastole; PWTs/d, posterior wall thickness in systole and diastole; SF (%), shortening fraction; SV, stroke volume.

### 2.2 Adult *Alms1* KO Rats (14–16 Weeks Old) Develop Hyperphagia and Obesity

**FIGURE 2:**
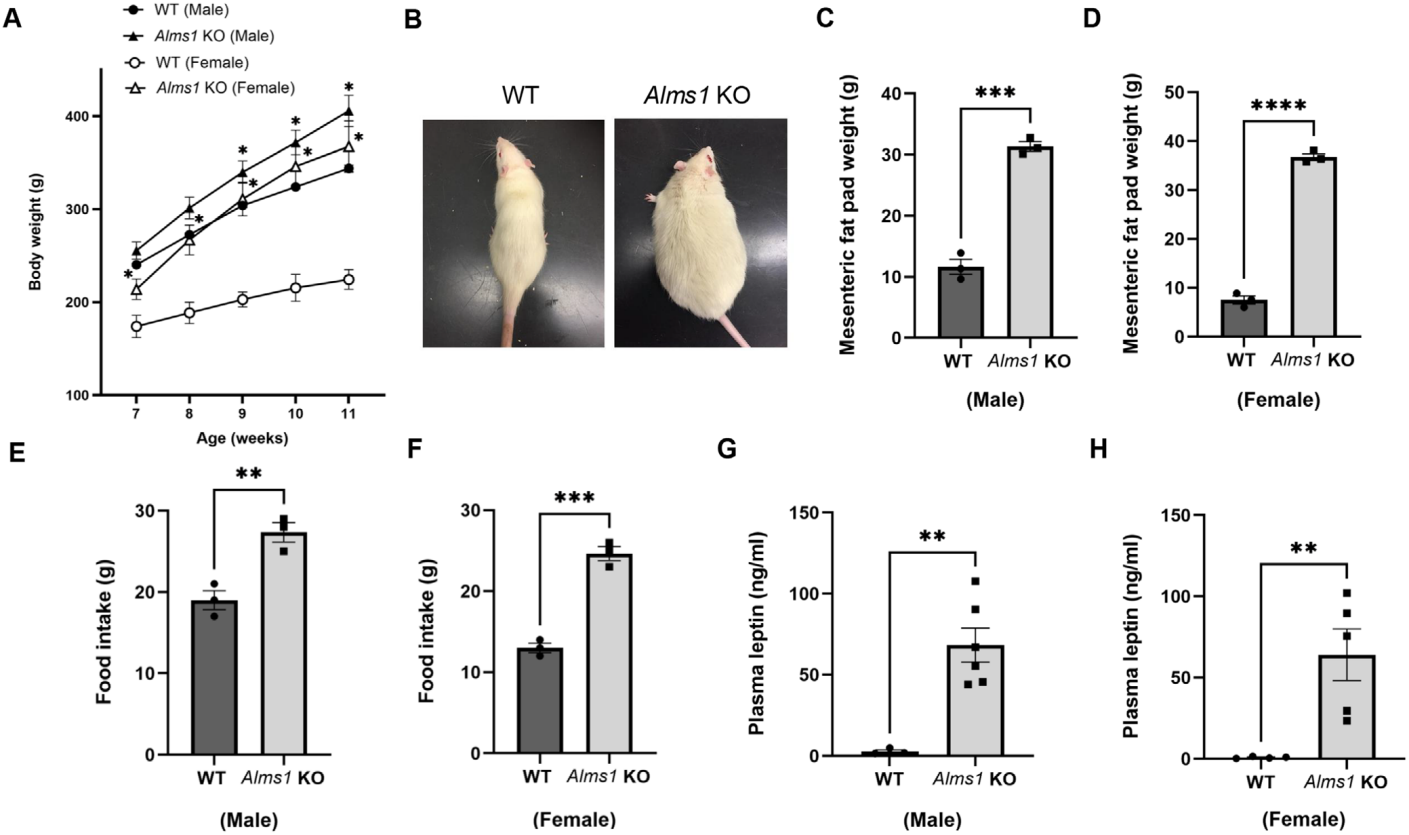
14–16 weeks old *Alms1* KO rats develop hyperphagia and obesity. (A) Body weight differences between the sexes of *Alms1* KO rats, *n* = 5 versus WT (*n* = 6). (B) Representative image of old female *Alms1* KO and WT rats. (C and D) Mesenteric fat pad weight in both sexes of *Alms1* KO rats, *n* = 3 versus WT. (E and F) Daily average food intake in both sexes of *Alms1* KO rats, *n* = 5 versus WT. (G and H) Plasma leptin concentration in both sexes of *Alms1* KO rats, *n* = 4 versus WT. Data is represented as mean ± SEM; analyzed by one or two‐tailed Student's *t*‐test; *p < 0.05, **p < 0.01 ***p < 0.0005.

To determine whether obesity develops later, we measured body weight in older *Alms1* KO rats. Our findings show that both female and male *Alms1* KO rats develop significant obesity, with males gaining excess weight starting at 9 weeks and females at 7 weeks (Figure 2A). In 16–18‐week‐old *Alms1* KO rats, mesenteric fat pad mass was significantly higher in both males (*Alms1* KO: 31.3 ± 3.4 g vs. WT: 11.36 ± 2.5 g) and females (*Alms1* KO: 35.8 ± 3.7 g vs. WT: 7.62 ± 1.8 g) compared to their WT counterparts (Figure 2C,D). Representative images of female *Alms1* KO rats at 16 weeks further confirm their obese phenotype (Figure 2B). To investigate the underlying cause of obesity, we measured food intake in 16–18‐week‐old *Alms1* KO rats and found that both males (*Alms1* KO: 27 ± 3 g/day vs. WT: 19 ± 2 g/day) and females (*Alms1* KO: 25 ± 2.5 g/day vs. WT: 13 ± 1 g/day) are hyperphagic (Figure 2E,F), suggesting increased food consumption as a potential driver of weight gain. Furthermore, plasma leptin levels were significantly elevated in 16–18‐week‐old *Alms1* KO rats, with males (male *Alms1* KO: 68.3 ± 10.5 vs. WT: 2.75 ± 1.1 ng/mL) and females (female *Alms1* KO: 64.1 ± 15.9 vs. WT: 0.9 ± 0.3 ng/mL) displaying severe hyperleptinemia (Figure 2G,H). These findings suggest that *Alms1* KO rats may develop progressive leptin signaling deficiencies, leading to blunted satiety and persistent hyperphagia, which are hallmark features of leptin resistance, suggestive of leptin resistance.

### 2.3 *Alms1* KO Rats Exhibit Altered Lipid Profile

**FIGURE 3:**
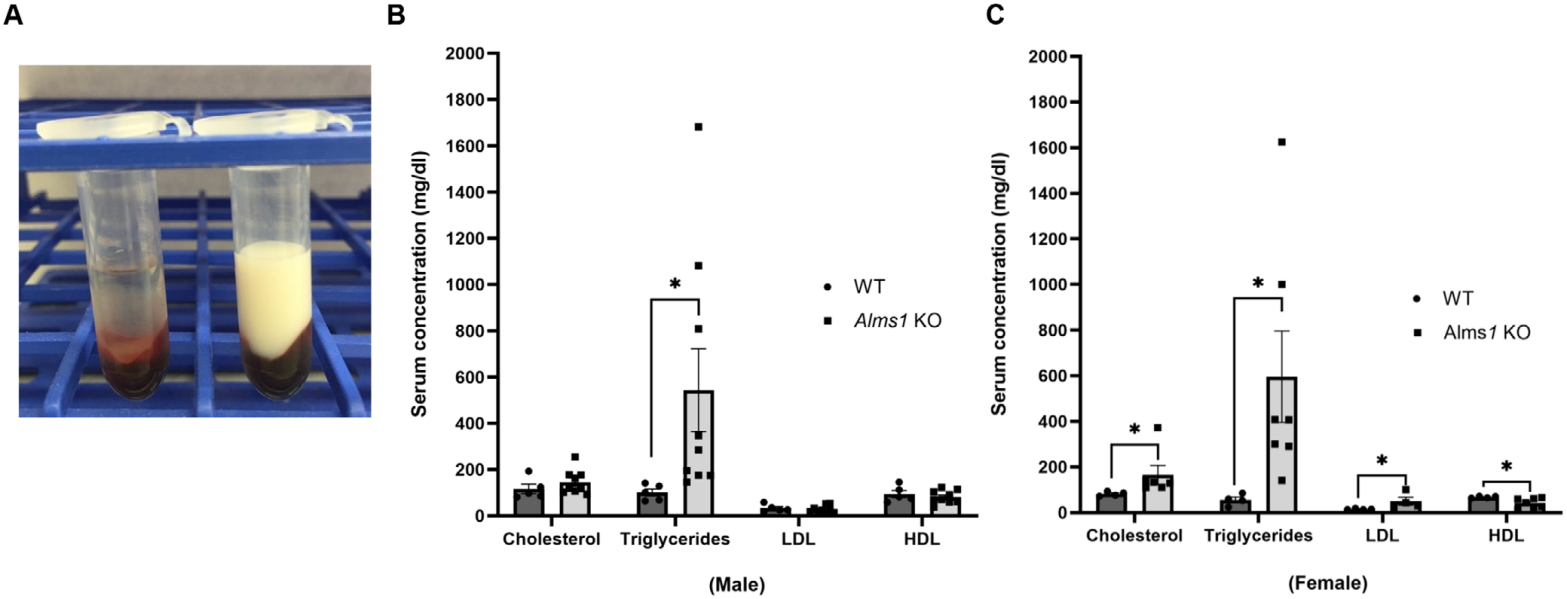
*Alms1* KO rats exhibit altered lipid profiles. (A) Representative image of blood collected from WT (left) and *Alms1* KO (right) rats. (B) Serum lipid profile from male *Alms1* KO: *N* = 7, WT: *N* = 4, *p < 0.01 versus WT and (C) Females *Alms1* KO: *N* = 7, WT: *N* = 4, *p < 0.05 versus WT. Data is represented as mean ± SEM; analyzed by a one‐tailed Student's *t*‐test.

Metabolic syndrome is commonly associated with abnormalities in blood lipid profiles, including serum triglycerides, low‐density lipoprotein (LDL), high‐density lipoprotein (HDL), and total cholesterol [4, 5, 6, 7]. In 16–18 weeks old *Alms1* KO rats, serum appeared milky, suggesting hyperlipidemia (Figure 3A). To confirm this, we measured serum lipid levels and found that triglyceride levels were significantly elevated in both males (*Alms1* KO: 543.7 ± 179.3 mg/dL vs. WT: 100.3 ± 15.5 mg/dL) and females (*Alms1* KO: 595.9 ± 200.1 mg/dL vs. WT: 55.6 ± 12.5 mg/dL). Interestingly, significant changes in cholesterol, HDL, and LDL levels were observed only in female *Alms1* KO rats, but not in males (Figure 3B,C): Total cholesterol: *Alms1* KO: 166 ± 41.5 mg/dL vs. WT: 81.9 ± 4.9 mg/dL; HDL: *Alms1* KO: 41.9 ± 7.9 mg/dL vs. WT: 67.8 ± 2.2 mg/dL; LDL: *Alms1* KO: 49.5 ± 17.6 mg/dL vs. WT: 15 ± 1.4 mg/dL. These findings indicate that *Alms1* KO rats develop progressive hyperlipidemia with severe alterations in total cholesterol, HDL, and LDL levels specifically in females. This suggests that ALMS1 may play a role in sex‐based differences in lipid metabolism, potentially influencing female susceptibility to dyslipidemia in metabolic syndrome.

### 2.4 *Alms1* KO Rats Exhibit Insulin Resistance

**FIGURE 4:**
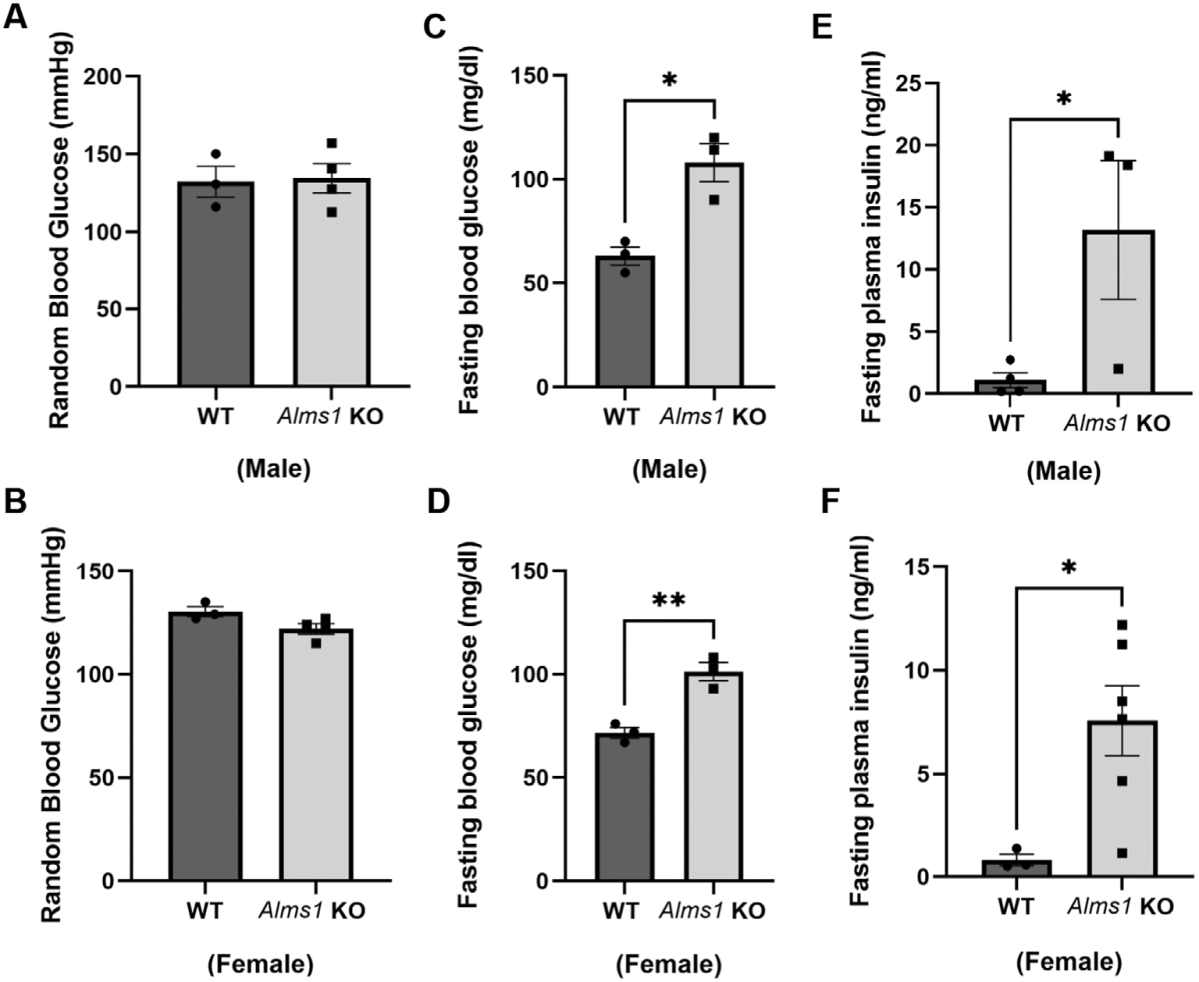
*Alms1* KO rats are insulin resistant. (A and B) Random blood glucose in male and female *Alms1* KO rats, *n* = 3, (C and D) Fasted‐blood glucose in male and female *Alms1* KO rats, *n* = 3, *p < 0.05 and (E and F) Plasma insulin in both sexes‐ male *Alms1* KO: *N* = 5 & WT: *N* = 3, female *Alms1* KO: *N* = 9 & WT: *N* = 6, *p < 0.05 versus WT. Data is represented as mean ± SEM; analyzed by one or two‐tailed Student's *t*‐test.

Insulin resistance is characterized by impaired insulin sensitivity, where cells lose the ability to effectively respond to insulin, leading to dysregulated glucose levels [1, 2, 3]. To determine whether *Alms1* gene deletion in rats results in insulin resistance—similar to what has been observed in *Alms1* mutant mice [13]—we assessed blood glucose and insulin levels. We first measured random glucose using tail snips and found no significant difference between *Alms1* KO and WT rats, in either males (*Alms1* KO: 134.5 ± 9.4 mg/dL vs. WT: 132.3 ± 9.9 mg/dL) or females (*Alms1* KO: 122 ± 2.5 mg/dL vs. WT: 130.3 ± 2.4 mg/dL) (Figure 4A,B). However, fasting blood glucose was significantly elevated in both males (*Alms1* KO: 108 ± 4 mg/dL vs. WT: 61 ± 17 mg/dL) and females (*Alms1* KO: 100 ± 12 mg/dL vs. WT: 71 ± 6 mg/dL) rat groups (Figure 4C,D). Additionally, fasting plasma insulin levels were markedly higher in *Alms1* KO rats (Male‐ *Alms1* KO: 13.2 ± 5.6 ng/mL vs. WT: 1.095 ± 0.6 ng/mL) and (Female‐ *Alms1* KO: 7.6 ± 1.7 ng/mL vs. WT: 0.82 ± 0.27 ng/mL) (Figure 4E,F), indicating hyperinsulinemia. Taken together, these findings suggest that *Alms1* KO rats exhibit impaired insulin signaling, resulting in hyperinsulinemia and elevated fasting glucose levels, key hallmarks of insulin resistance.

### 2.5 Male and Female *Alms1* KO Rats Develop Hypertension

**FIGURE 5:**
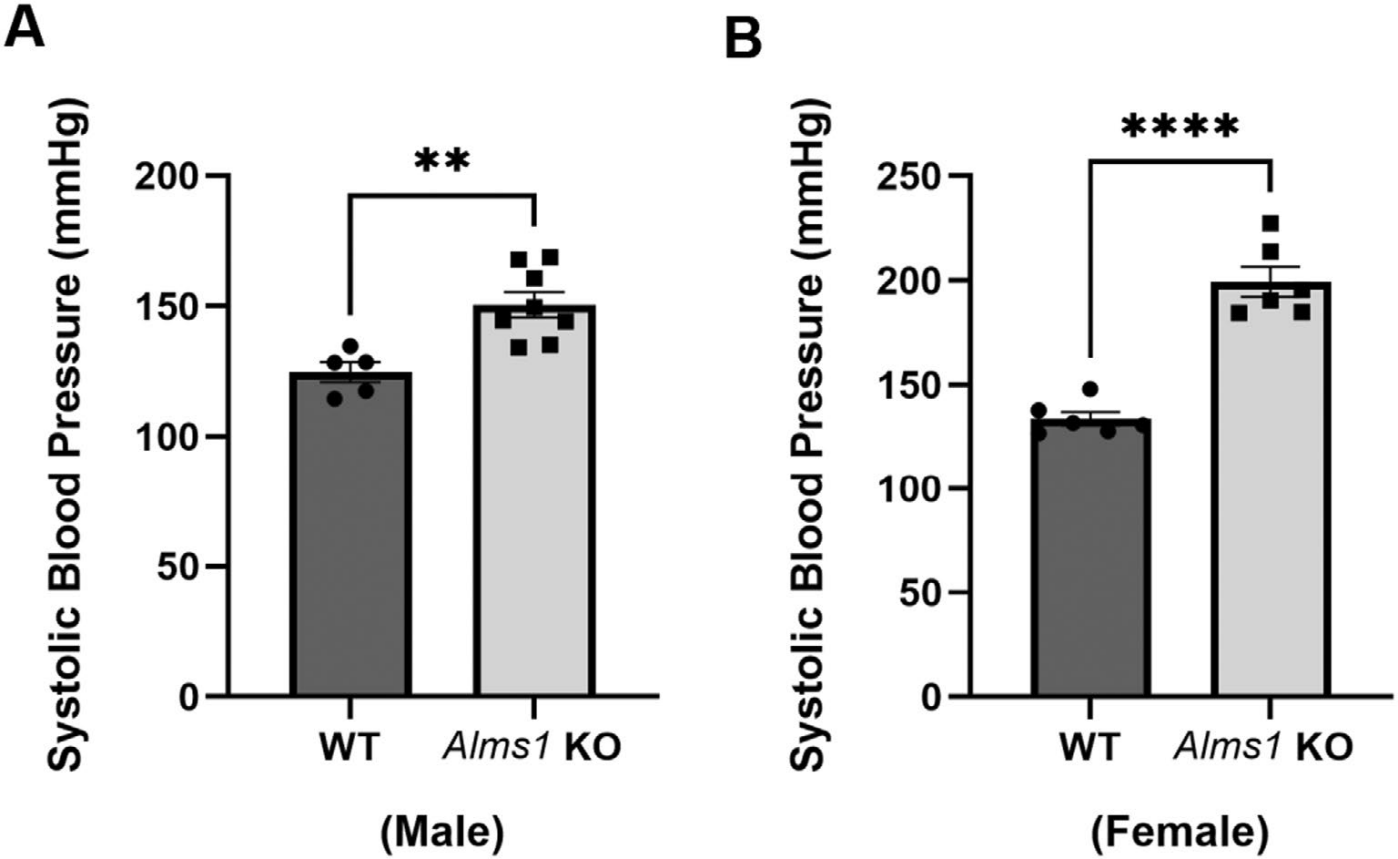
*Alms1* KO rats are hypertensive. (A) Systolic blood pressure in male *Alms1* KO rats‐ WT: *N* = 5 and *Alms1* KO: *N* = 8, **p < 0.005 versus WT. (B) Systolic blood pressure in female ALMS1 KO rats: *N* = 6, ****p < 0.0001 versus WT. Data is represented as mean ± SEM; analyzed by two‐tailed Student's *t*‐test.

Hypertension is a key risk factor that contributes to the severity of metabolic syndrome [17, 18]. To determine whether genetic deletion of *Alms1* leads to sex‐dependent increases in blood pressure, we measured systolic blood pressure (SBP) in 7–9‐week‐old *Alms1* KO rats and WT littermates on a regular salt diet (0.22% Na) using tail‐cuff plethysmography. Our findings indicate that both male and female *Alms1* KO rats are hypertensive, with SBP significantly elevated compared to WT: Males (*Alms1* KO: 151 ± 5.1 mmHg vs. WT: 125 ± 4.2 mmHg); Females (*Alms1* KO: 199 ± 7.1 mmHg vs. WT: 134 ± 3.3 mmHg). *Alms1* KO rats are hypertensive (Figure 5A).

Notably, SBP was significantly higher in female *Alms1* KO rats compared to their male counterparts (Figure 5B), suggesting that hypertension is more pronounced in females. This finding is particularly interesting given that previous studies have shown that female Dahl SS rats are typically protected from hypertension compared to males [19]. However, in *Alms1* KO rats, this female protection is lost, indicating that ALMS1 deletion abolishes the sex‐based resistance to hypertension observed in Dahl SS females.

### 2.6 *Alms1* KO Rats Exhibit Vascular Dysfunction

**FIGURE 6:**
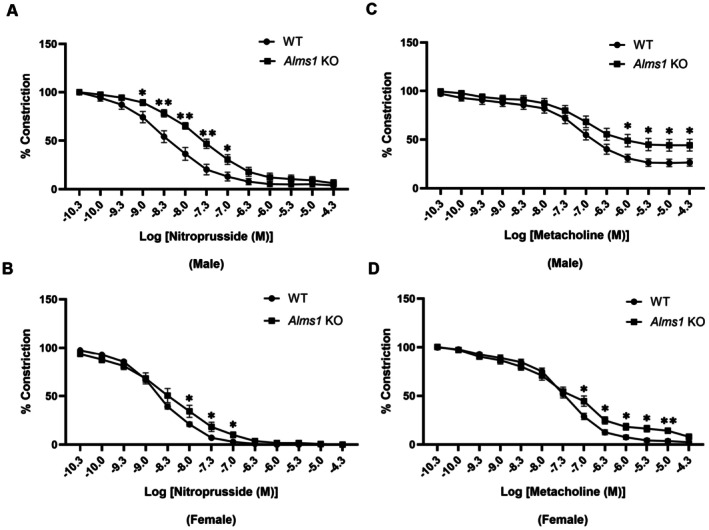
*Alms1* KO rats exhibit vascular dysfunction. (A and B) Nitroprusside‐induced vasorelaxation in thoracic aorta from 14 to 20 weeks‐old male and female *Alms1* KO rats; *n* = 6, *p < 0.05, **p < 0.005. (C and D) Methacholine‐induced vasorelaxation in thoracic aorta from 14 to 20 weeks‐old male and female *Alms1* KO rats; *n* = 6, *p < 0.05, **p < 0.005. Data is represented as mean ± SEM; analyzed by student's *t*‐test.

The role of ALMS1 in vascular reactivity is unkown. However, each component of the metabolic syndrome has been shown to impair vascular endothelium and smooth muscle cell function, leading to vascular dysfunction and disrupted vascular homeostasis [20]. Given the metabolic abnormalities observed in *Alms1* KO rats, we investigated whether *Alms1* genetic deletion affects vascular function. To assess vascular reactivity, we measured the vasorelaxation responses of thoracic aortic rings isolated from WT and *Alms1* KO rats to methacholine (endothelium‐dependent relaxation) and nitroprusside (endothelium‐independent relaxation via nitric oxide donor (NO)) [21]. Since older rats exhibit significant metabolic dysfunction, vascular reactivity was tested in 14–20 weeks‐old rats. Our findings show that thoracic aortas from both male and female *Alms1* KO (14–20 weeks old) exhibited reduced vasorelaxation in response to nitroprusside (Figure 6A,B). This indicates impaired smooth muscle relaxation in response to NO donors, suggesting a defect in arterial smooth muscle function. Notably, male *Alms1* KO rats exhibited this impairment at lower nitroprusside doses than females, indicating a more pronounced vascular dysfunction in males. Additionally, thoracic aortic rings from both female and male *Alms1* KO rats displayed significantly reduced endothelium‐dependent relaxation to methacholine (Figure 6C,D), further supporting endothelial dysfunction in *Alms1* KO rats. Overall, these findings suggest that ALMS1 plays a critical role in maintaining vascular function, and its deletion leads to both endothelial and smooth muscle dysfunction, in the presence of metabolic abnormalities.

### 2.7 *Alms1* KO Rats Exhibit Cardiac Dysfunction

**FIGURE 7:**
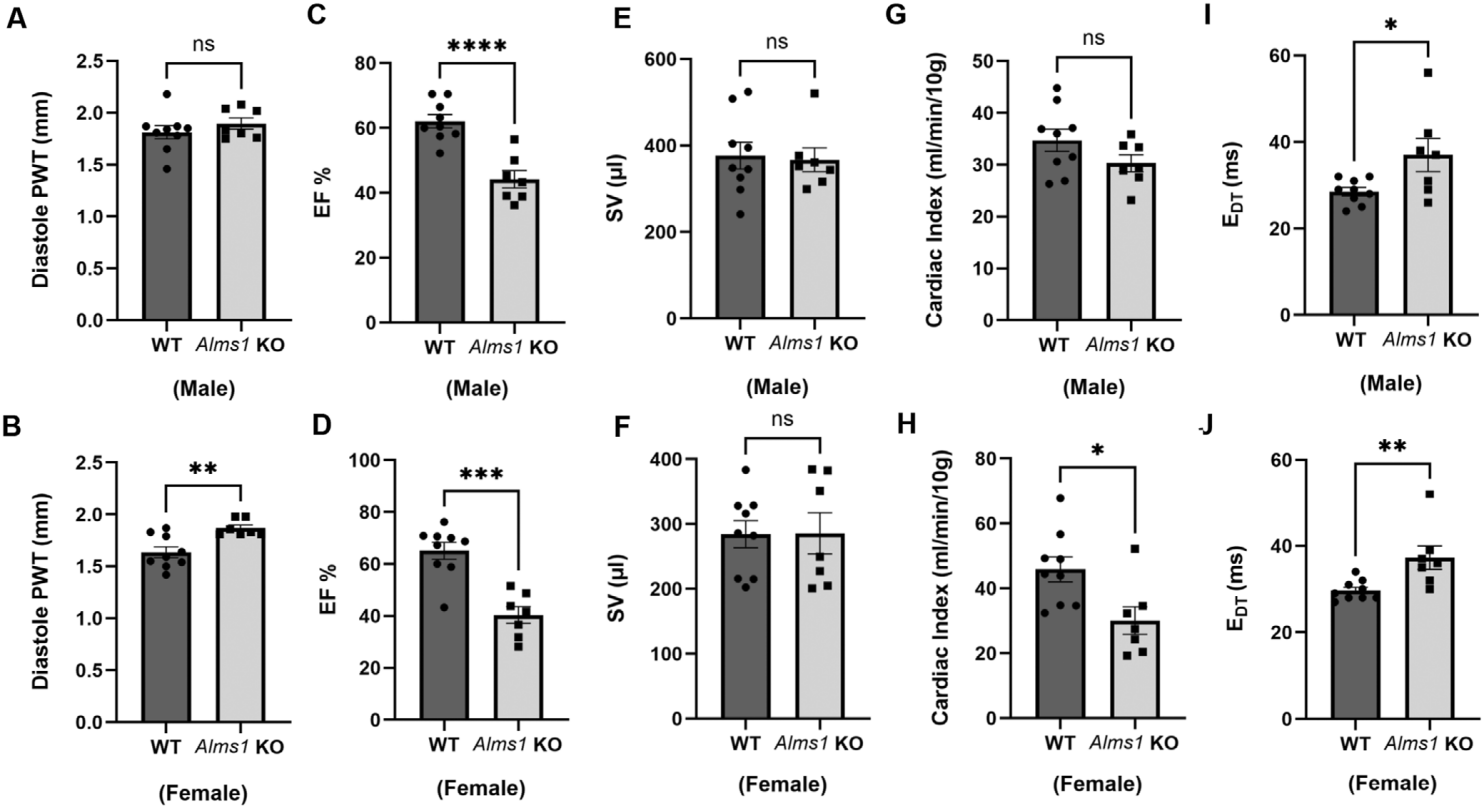
*Alms1* KO rats exhibit diastolic dysfunction. (A and B) Diastolic posterior wall thickness (PWT) in 14–16‐week‐old male and female *Alms1* KO rats. (C and D) Ejection Fraction (EF%) in 14–16‐week‐old male and female *Alms1* KO rats. (E and F) Stroke Volume (SV) in 14–16‐week‐old male and female *Alms1* KO rats. (G and H) Cardiac Index CI = cardiac output/10 g in 14–16‐week‐old male and female *Alms1* KO rats. (I and J) E_DT_ (deceleration time of the early mitral filling wave) in 14–16‐week‐old male and female *Alms1* KO rats. WT: *N* = 7; *Alms1* KO: *N* = 9. *p < 0.05, **p < 0.01, ***p < 0.001, ****p < 0.0001 versus WT.

Echocardiographic analysis (Table 2, Figure 7) revealed age‐ and sex‐dependent structural and functional cardiac changes in *Alms1* KO rats. At 6–7 weeks of age, systolic function was preserved in both sexes, with no significant differences in ejection fraction or shortening fraction compared to WT controls (Table 2). Interventricular septal thickness in systole and diastole did not differ between genotypes at this early age. However, posterior wall thickness in diastole was modestly but significantly increased in young female *Alms1* KO rats, indicating the presence of early, sex‐specific morphological remodeling despite preserved systolic performance (Table 2).

By 14–16 weeks of age, *Alms1* KO rats exhibited marked cardiac remodeling and dysfunction. Interventricular septal thickness was significantly increased in older females, but not males, indicating sex‐dependent septal remodeling (Figure 7A,B; Table 2). This reflects early ventricular remodeling, possibly as a compensatory response to progressive functional decline (Figure 7C,D). Similarly, posterior wall thickness in diastole was significantly increased in older female *Alms1* KO rats, reflecting progressive structural remodeling accompanying declining cardiac function. Left ventricular dimensions and areas, both in systole and diastole, were significantly increased in *Alms1* KO rats, consistent with LV dilation. Despite these geometric changes, LV mass normalized to body weight remained unchanged, indicating eccentric remodeling rather than hypertrophy.

Functionally, stroke volume was not significantly different between genotypes, suggesting that ventricular dilation did not translate into enhanced preload reserve or effective contractile compensation, suggesting limited contractile reserve despite chamber enlargement (Table 2, Figure 7E,F). Cardiac index was significantly reduced in older female *Alms1* KO rats, reflecting impaired cardiac output relative to metabolic demand (Figure 7G,H). Diastolic function was also compromised, as evidenced by a significant prolongation of early mitral inflow deceleration time (Figure 7I,J), whereas E/A ratio remained unchanged, suggesting impaired relaxation with preserved early filling dynamics (Table 2). Collectively, these findings demonstrate a progression from subtle, early structural alterations to overt LV dilation, systolic dysfunction, and impaired diastolic relaxation with aging, underscoring a critical role for ALMS1 in maintaining cardiac structure and function.

## 3 Discussion

The coexistence of hypertension and diabetes mellitus triples the risk for cardiovascular disease, as indicated in the Framingham Heart study [17] and MRFIT trials [18]. Obesity‐related hypertension is also known to predispose to coronary heart disease, renal dysfunction, left ventricular hypertrophy, and congestive heart failure [22, 23, 24, 25]. The presence of hypertension in the setting of obesity and diabetes creates a vicious feed‐forward loop that also increases the occurrence of end‐stage kidney failure [26]. Despite the clinical significance of metabolic syndrome, there are few animal models that fully replicate all its hallmarks, including hypertension. In this study, we demonstrate that genetic deletion of *Alms1* in rats results in a comprehensive metabolic syndrome phenotype, including obesity, insulin resistance, hyperlipidemia, and hypertension, without dietary intervention and independent of sex. Moreover, growing evidence links ALMS1 to metabolic disorders, hypertension, and kidney disease in the general population, further supporting its role as a key regulator of cardiometabolic health [10, 27, 28, 29, 30]. For example, two independent genome‐wide association (GWAS) studies have identified single nucleotide polymorphisms (SNPs) in the *Alms1* locus that are associated with lower GFR and an increased risk of chronic kidney disease [27, 28, 29, 30]. Quantitative trait loci (QTL) linked to blood pressure and salt‐sensitive hypertension have been mapped to the *Alms1* locus [31, 32, 33, 34, 35]. Importantly, patients lacking functional ALMS1 exhibit severe manifestations of metabolic syndrome including progression of cardiac and kidney disease [4, 5, 6, 7, 8, 9, 10, 36, 37, 38]. This suggests that ALMS1 plays a critical role in regulating pathways involved in metabolism and cardiovascular function, a conclusion further supported by our findings.

A key finding from our studies is that the *Alms1* KO rat closely models the development of metabolic syndrome. Specifically, 14–20 weeks‐old male and female *Alms1* KO rats exhibit hyperinsulinemia, obesity, hyperphagia, hypertriglyceridemia and hyperleptinemia. Also, female *Alms1* KO rats display more severe metabolic abnormalities, including higher serum cholesterol, LDL levels, along with reduced HDL levels compared to males. Insulin resistance in *Alms1* KO rats may develop secondarily to obesity, since we found that young (6–9 weeks) nonobese *Alms1* KO rats do not exhibit hyperinsulinemia [11]. In most groups studied, a significant increase in body weight was statistically significant in 6–7‐week‐old rats (Table 2), supporting that *Alms1* deletion promotes early onset weight gain. However, there was some variability on the onset of statistically significant body weight gain, in part due to variability in the control groups (Table 1). Regardless, after 6–7 weeks of age, Alms1 KO rats were heavier or tended to be heavier in all groups studied. Importantly, hyperleptinemia is unlikely to be secondary to obesity, because 7‐week‐old *Alms1* KO rats exhibit elevated plasma leptin levels before the onset of obesity. This suggests that ALMS1 may play a primary role in leptin production or signaling leading to primary hyperleptinemia, subsequent leptin resistance and hyperphagia in *Alms1* KO rats. Our data in rats is consistent with results from mouse models of ALMS1 deficiency. In foz/foz mice, the loss of Alms1 induces hyperleptinemia, hyperphagia and obesity. In other Alms1 KO mouse models, global or mesenchymal stem cell‐specific *Alms1* deletion induces elevated serum leptin concentrations [14]. Together these findings suggest that *Alms1* plays a crucial role in the control of leptin release and satiety regulation [15]. Unfortunately, neither blood pressure, vascular reactivity nor cardiac function were studied in *Alms1* mutant or KO mice prior to our study. Overall, our study establishes *Alms1* KO rats as an excellent model for investigating both, (A) the mechanisms underlying metabolic dysfunction secondary to hyperleptinemia and (B) cardiovascular dysfunction caused by loss of *Alms1* or secondary to the obesity caused by Alms1 deletion. This rat model will also be useful to studying new therapeutic interventions targeting obesity and MetS.

ALMS1 has been implicated in ciliary function, but its role in cilia biology appears to be cell‐type specific. A study using mouse inner medullary collecting duct (mIMCD3) cells demonstrated that ALMS1 knockdown resulted in defective ciliogenesis [39]. However, renal tubules from *Alms1* mutant mice displayed normal cilia assembly [13], indicating that ALMS1 may not be essential for ciliogenesis in all cell types. Similarly, fibroblasts from Alström syndrome patients exhibit normal cilia morphology but show defective membrane trafficking [40], suggesting that the pathology observed in these patients may stem from intracellular trafficking defects rather than primary cilia dysfunction. Our previous data indicate that *Alms1* KO rats exhibit normal ciliary development in renal tubules [11], however, we only measured cilia length and number, not cilia‐dependent signaling pathways [11]. Despite the apparently normal cilia structure, vascular ciliary function such as flow‐induced nitric oxide synthesis [13] may be impaired in *Alms1* KO rats, potentially contributing to the defective Ach‐induced vasorelaxation observed in this study. Further studies are needed to determine the molecular mechanisms by which ALMS1 regulates endothelial‐mediated vasodilation and whether it involves nitric oxide synthase (eNOS) and nitric oxide (NO) signaling in large and resistance arteries. Understanding these pathways could provide new insights into ALMS1's role in vascular and heart function and metabolic disease.

We previously demonstrated that ALMS1 plays a role in regulating renal function [11, 41] and here we showed a role in cardiac and vascular function. However, more is known about the role of Alms1 in metabolism and energy homeostasis. Defective ALMS1 expression/signaling is involved in obesity pathogenesis, as Alström syndrome patients develop early‐onset obesity, and *Alms1*‐targeted mutations in mice cause progressive obesity, insulin resistance, diabetes, and hepatic steatosis by 18–21 weeks of age [13]. In the foz/foz mouse strain, which carries a mutation in *Alms1*, obesity is driven by hyperphagia, and impaired brown adipose tissue (BAT) diet‐induced thermogenesis [42]. Studies using 3 T3‐L1 preadipocytes showed that stable knockdown of ALMS1 impairs lipid accumulation and reduces adipocyte gene expression following hormonal induction of adipogenesis [43], suggesting that partial defects in early adipogenesis may contribute to the severity of obesity in Alström syndrome [42, 44]. However, pre‐adipocytes isolated from *Alms1* mutant mice exhibited normal adipogenic differentiation and developed into mature adipocytes with reduced insulin‐stimulated glucose uptake. Also, *Alms1*‐deficient mice display decreased insulin‐dependent GLUT4 translocation to the plasma membrane in adipose tissue [12]. This defect may contribute to glucose intolerance and compensatory hyperinsulinemia in Alström patients and *Alms1* transgenic mouse models [13]. Interestingly, caloric restriction in an Alström patient prevented hyperinsulinemia [45], supporting the idea that hyperinsulinemia may develop secondary to obesity, consistent with our data in *Alms1* KO rats. In addition, studies in a new Alström syndrome mouse model “fat aussie” suggest a critical role for ALMS1 in obesity and diabetes [16]. These observations further suggest that adipose tissue with impaired glucose uptake may compensate for de novo lipogenesis, leading to excessive fat accumulation and obesity [16, 45]. Together, these observations support a key role for ALMS1 in adipose tissue function, glucose metabolism, and obesity development. Understanding ALMS1's role in adipogenesis, insulin signaling, and lipid metabolism could provide new insights into the pathophysiology of metabolic syndrome and potential therapeutic strategies.

Sex hormones play a critical role in regulating plasma lipid metabolism and contribute to sexual dimorphism in plasma lipid profiles, which may partially explain the cardiovascular protective effects in females [1, 2, 3, 17, 18]. Several genes have been mechanistically linked to sex‐based differences in the development of metabolic syndrome [1, 2, 3, 12, 16]. Some sex‐based differences in metabolic disorders have also been observed in a mouse model of *Alms1* mutation [13]. However, only male *Alms1* mutant mice became hyperglycemic [13]. Notably, that study did not characterize sex‐based phenotypic differences in hypertension, vascular, or cardiac function associated with ALMS1 loss [13]. We previously showed that young *Alms1* KO rats have elevated blood pressure, which preceded obesity [11]. We found higher surface expression of NKCC2 and elevated NKCC2‐mediated NaCl transport in the kidney of *Alms1* KO rats. As shown here, male *Alms1* KO were hypertensive before 10 weeks of age, but the hypertension observed is not likely related to elevated renin release, since plasma renin activity (PRA) was not different between the strains [11]. We hypothesize that the hypertension in *Alms1* KO rats is, in part, dependent on impaired pressure‐natriuresis [19] and decreased endothelial‐mediated vasorelaxation, but there are likely to be additional mechanisms. Here, we show that female *Alms1* KO rats exhibited a more severe form of hypertension and metabolic syndrome than males, indicating a potential sex‐specific vulnerability to ALMS1. The underlying cause of these sex differences remains unclear. However, ours and other studies identified that ALMS1 interacted with several proteins associated with estrogen receptor signaling such as prohibitin and integrin linked kinase (ILK) [46, 47]. This suggests that ALMS1 may play a role in estrogen‐mediated metabolic regulation; however, further studies are needed to explore the potential role of ALMS1 in estrogen signaling, sex‐hormone levels, and to determine why worse outcomes are observed in females.

This study demonstrates that ALMS1 deficiency in rats leads to progressive cardiac dysfunction, affecting both systolic and diastolic function and driving structural remodeling. Echocardiographic data reveal that *Alms1* KO rats exhibit age‐related left ventricular (LV) dilation, increased wall thickness, and reduced shortening/ejection fraction (SF/EF), all indicative of impaired systolic function [48, 49]. While E/A ratio was slightly reduced in *Alms1* KO rats, it was not statistically significant. In contrast, prolonged E_DT_ indicates impaired ventricular relaxation, suggesting early diastolic dysfunction may be more sensitively captured by E_DT_ than E/A ratio in this model [50, 51]. Notably, these cardiac impairments occurred independently of sex, aligning with previous findings showing severe systolic and/or diastolic dysfunction in both male and female obese rats. This contrasts with earlier studies suggesting that female rodents are typically protected from cardiac injury and dysfunction due to hypertension or myocardial infarction [52, 53, 54]. Further research is needed to elucidate the mechanisms underlying the absence of cardiac protection in *Alms1* KO females and determine whether sex hormones, metabolic disturbances, or cilia‐related pathways contribute to their heightened susceptibility to cardiac dysfunction.

Hypertension is likely to play a role in the cardiac remodeling observed in *Alms1* KO rats. Chronic elevation of arterial pressure increases left ventricular afterload, promoting early increases in wall thickness as an adaptive response to normalize wall stress. With sustained pressure overload and progressive metabolic stress, this compensatory remodeling appears to transition toward maladaptive eccentric remodeling [55, 56], characterized by left ventricular dilation, increased chamber volumes, and declining systolic performance. Despite ventricular dilation, stroke volume remained unchanged, suggesting that increased preload does not translate into effective augmentation of forward output, due to impaired contractile reserve and persistent afterload excess. This concept is consistent with classic experimental and clinical studies showing that chronic hypertension leads to preserved stroke volume early on, followed by ventricular dilation and systolic dysfunction as afterload mismatch and myocardial stress progress. Thus, in *Alms1* KO rats, hypertension acts both as an initiating and amplifying factor in the evolution of cardiac hypertrophy, chamber dilation, and functional decline.

The hypertrophic changes observed in *Alms1* KO rats—including interventricular septal thickness (IVST) and posterior wall thickness (PWT), particularly at 14–16 weeks, likely represent an adaptive response to chronic pressure overload in the setting of ALMS1 deficiency. However, this maladaptive remodeling, characterized by cardiac dilation, closely resembles the cardiomyopathy patterns associated with Alström syndrome and is linked to an increased risk of heart failure [57, 58, 59]. Importantly, our findings also indicate that ALMS1 deficiency initiates subtle morphological alterations at an early age. Younger *Alms1* KO rats exhibited changes in septal and posterior wall thickness despite preserved systolic function. These early morphological changes may represent the first stage in a continuum of maladaptive remodeling. With aging, these alterations progress to overt LV dilation and functional impairment, highlighting a temporal link between early structural remodeling and later development of heart failure phenotypes in *Alms1* KO rats.

Additionally, reduced systolic function in *Alms1* KO rats suggests impaired contractility, possibly due to disrupted interactions between ALMS1 and structural proteins such as ACTN4, which are essential for maintaining cytoskeletal stability in cardiomyocytes [60, 61, 62]. The cardiac dysfunction observed in our experimental models closely mirrors the human phenotype of Alström syndrome, in which early‐ and late‐onset cardiomyopathy with systolic impairment, abnormal myocardial strain, and fibrotic remodeling are prominent clinical features, thereby reinforcing a direct and heart‐intrinsic role for ALMS1 in myocardial homeostasis [10]. Although genetic deletion of ALMS1 clearly impacts cardiac function, it remains uncertain whether these effects arise directly from ALMS1's role in cardiomyocytes or indirectly through vascular dysfunction. ALMS1 is known to be expressed in cardiomyocytes, and its loss may impair contractility, calcium handling, or other mechanisms essential for normal heart function. However, some of the observed cardiac dysfunction may be secondary to hypertension, obesity, or dyslipidemia in these animals.

There are some limitations of our study that should be discussed and will be further explored in the future. One of them is that we have not been able to measure body composition. Obesity and fat deposits were measured after dissection of mesenteric fat depots and there is a clear difference. However, we do not know if there are differences in lean mass in *Alms1* KO rats. Since rats are of different sizes, linear regression/ANCOVA should ideally be used to normalize food intake and other parameters by lean mass. The deletion of *Alms1* was done in rats of the Dahl salt‐sensitive genetic background. We thought this was positive since these rats are prone to develop hypertension and we wanted a model that better matches metabolic syndrome and Alström syndrome. It is unclear whether deletion of *Alms1* in salt‐resistant rats, such as the Sprague Dawley rat, would cause hypertension. An additional limitation is that we have not been able to quantify the whole repertoire of symptoms observed in Alström syndrome [13, 63]. We observed loss of vision, as examined by our vet team, in some but not all *Alms1* KO rats, but we have not quantified loss of hearing or vision, which are reported in some, but not all, patients with Alström syndrome [63]. In patients, there is variability in the incidence of symptoms. Most likely, because there are hundreds of different single nucleotide variants that cause loss of ALMS1, as per Genome Wide Association Study catalogue (https://www.ebi.ac.uk/gwas). These single nucleotide variants are associated with different traits such as abnormal urinary metabolite levels in chronic kidney disease [64], serum metabolite levels associated with coronary heart disease and type 2 diabetes [65], suggesting that different portions of the gene/protein may still have function even when truncated, or there are splice variants, or differential expression levels.

To conclude, the *Alms1* KO rat model serves as a valuable model for metabolic syndrome, exhibiting both vascular and cardiac impairments that could aid in drug target discovery and therapeutic development. These findings underscore ALMS1's essential role in maintaining cardiac structure and function and suggest that ALMS1 deficiency may predispose individuals to heart failure. Further research is needed to identify the specific molecular pathways through which ALMS1 preserves cardiac integrity and to evaluate targeted therapies for ALMS1‐related cardiac remodeling and dysfunction.

## 4 Methods

### 4.1 Generation of *Alms1* KO Rats

Alms1 KO rats were generated by the Gene Editing Rat Resource Center (GERRC, http://rgd.mcw.edu/wg/custom_rats/gerrc). Frameshift deletion of 17 bp in exon 1 (containing a Nci1 restriction site) of *Alms1* gene was facilitated by injecting zinc Finger nucleases targeting the sequence CCCGCCTCCGACTCCGCCtccgtcCTCCCGGCACCAGTA into Dahl SS/JrHsdMcwi rat embryos. This deletion caused a frameshift leading to a pre‐mature stop codon in exon 2. *Alms1* KO homozygous and WT rats were genotyped by Transetyx Inc. using Taqman PCR (not conventional PCR) using custom designed oligonucleotide sequences to be specific to genomic DNA (Figure 1A,B). Transnetyx uses a high‐throughput, real‐time PCR‐based system for genotyping. The TaqMan assays target specific sets of DNA sequences, generating millions of copies of the target DNA molecule analyzed in a real‐time fluorescent readout throughout the reaction as it occurs. Transnetyx designed a specific set of primers for rat ALMS1 based on the frameshift mutation in exon 1.

Male and female *Alms1* KO rats were generated in a Dahl salt‐sensitive background. Wild type (WT) age‐matched littermate controls were male and female Dahl salt‐sensitive rats. Animals were fed a standard diet (0.22% Na, 1% K) from Envigo (Indianapolis, IN). All procedures involving live animals were approved by the Institutional Animal Care and Use Committee (IACUC) of Henry Ford Hospital and conducted following guidelines.

### 4.2 Blood Pressure Measurements

Systolic blood pressure was also measured in awake rats by tail‐cuff plethysmography. Rats were trained for over 2 weeks, 4 measurements/week starting exactly at the same time of day (10 am EST), acclimated to 37°C in the Kent Scientific Coda High Throughput system for 30 min before acquiring the reported SBP measurements. The readings for the last 3 days of measurement are similar to the ones reported for the last day indicating the rats have acclimated. SBP was measured in 2 automated cycles per rat. Each cycle consisted of 10 measurements.

### 4.3 Metabolic Cage Studies

Food intake was measured using metabolic cages; food left over at 24 h was subtracted from food given initially at time 0 to get the food consumed per 24‐h period.

### 4.4 Plasma Insulin and Leptin Measurement

Whole blood was collected from the thoracic aorta of rats anesthetized with Pentobarbital (Nembutal; 50 mg/kg IP) and was immediately mixed with an appropriate volume of EDTA (5:1 ratio) to prevent blood coagulation. Blood was then spun at 200 g for 20 min to collect plasma. Using an enzyme immunoassay kit from Cayman (Ann Arbor, MI), plasma samples were used to measure insulin and leptin concentration.

### 4.5 Blood Glucose and Serum Lipid Profiling

Random and fasting blood glucose was measured using the Wavesense Presto Blood Glucose meter (AgaMatrix, Salem, NH). Lipid profiling using rat serum samples was done using Siemens healthcare diagnostic analyzer (Siemens, Malvern, PA) according to the manufacturer's instructions.

### 4.6 Vascular Reactivity

Briefly, rats were anesthetized with pentobarbital (50 mg/kg; IP). Thoracic aortas were carefully excised from euthanized rats, cleaned of connective tissue and fat, and cut into 3‐mm‐long ring segments. The aortic rings were mounted on stainless steel pins in a 4‐channel DMT 620 M myograph system (Danish Myo Technology, Aarhus, Denmark) filled with 10 mL of warmed, aerated physiological salt solution (PSS; 130 mM NaCl, 4.7 mM KCl, 1.17 mM MgSO_4_, 1.18 mM KH_2_PO_4_, 14.9 mM NaHCO_3_, 1.6 mM CaCl_2_, 5.5 mM glucose, and 0.026 mM EDTA), maintained at 37°C and bubbled continuously with 95% O_2_ and 5% CO_2_. After a 30‐min equilibration period, the rings were gradually stretched to a resting passive tension of 1 g in 0.3 g increments every 15 min to approximate optimal length‐tension relationships. Following this normalization procedure, vascular integrity and maximal contractile capacity were confirmed by stimulation with 60 mM K^+^ PSS, and only vessels responding with a robust contraction were used for further analysis. After washout and re‐equilibration, cumulative concentration‐response curves to phenylephrine (PE; 10^−10^ to 10^−5^ M) were generated to assess contractile responses. In a separate set of experiments, aortic rings were precontracted with a submaximal concentration of PE (about EC_80_), and endothelium‐dependent relaxation was assessed by cumulative addition of methacholine (10^−10^ to 10^−5^ M). To evaluate endothelium‐independent relaxation, another set of PE‐preconstricted rings were challenged with sodium nitroprusside (SNP; 10^−10^ to 10^−5^ M). Tension changes were continuously recorded and analyzed using LabChart software (ADInstruments). Vasoconstrictor responses were expressed as a percentage of the maximal contraction to 60 mM K^+^, while vasorelaxation responses were expressed as a percentage of PE‐induced precontraction.

### 4.7 Echocardiography

After anesthetizing the rats (pentobarbital, 50 mg/kg; IP), transthoracic echocardiography and Doppler sonography using an Acuson Sequoia C 256 with a 15‐MHz transducer were performed together with electrocardiography in female and male WT and *Alms1* KO rats at 6‐weeks and 14‐to‐16‐weeks of age. Electrocardiography was simultaneously recorded to synchronize image acquisition. M‐mode echocardiography was initially performed in the parasternal long‐axis view to measure left ventricular (LV) internal dimensions at end‐diastole and end‐systole (LVIDd, LVIDs), and subsequently in the short‐axis view at the level of the papillary muscles to calculate LV ejection fraction (LVEF) using the Teichholz formula. For diastolic function assessment, pulse wave Doppler was acquired from the apical four‐chamber (A4C) view by aligning the Doppler cursor parallel to transmitral inflow. Early (E) and late (A) diastolic filling velocities and the E/A ratio were calculated from at least 5–7 cardiac cycles per rat at a sweep speed of 200 mm/s. All Doppler recordings were performed at end‐expiration to minimize respiratory variability.

Stroke volume was estimated from aortic root diameter (AoD) and velocity‐time integral (VTI) using the formula:

$$\text{Stroke Volume}=\text{𝜋}\times {\left(\mathrm{AoD}/2\right)}^{2}\times \mathrm{VTI}$$

Cardiac index (CI) was then calculated as:

$$\mathrm{CI}=\left(\text{Stroke Volume}\times \text{Heart Rate}\right)/10\;g\;\mathrm{BW}$$

Data acquisition and measurements were performed using native Acuson Sequoia software with built‐in calipers. All values were averaged over 3–5 cardiac cycles, and analyses were conducted by blinded investigators. The Doppler and echocardiographic protocols followed our previously published methods [66, 67, 68].

### 4.8 Statistical Analysis

Results are expressed as mean ± SEM. Single intergroup comparisons between two groups were performed with a Student's *t*‐test using GraphPad Prism software. *p* < 0.05 was considered statistically significant.

### 4.9 Inclusion and Exclusion Criteria

No outliers were excluded from the study. Blinding: In vascular reactivity experiments, the experimenter was blinded to the identities of rats. Power analysis: Based on previous experience, animal cohorts of 3–6 rats per group are deemed sufficient to detect differences between groups with a 90% power and a 5% type I error rate.

### 4.10 Data Sharing

We will follow all NIH policies concerning sharing reagents, materials, and information with other investigators. Detailed protocols are provided to everyone who requests them. Upon publication, this manuscript will be submitted to the National Library of Medicine's PubMed Central as outlined by NIH policy.

## Author Contributions

**Ankita B. Jaykumar:** conceptualized, supervised, designed and performed experiments, performed analysis, wrote the manuscript, generated initial figures; **Sumit R. Monu:** collected and analyzed data; **Nour‐Eddine Rhaleb:** performed vascular experiments, analyzed echocardiography data and edited the manuscript; **Xiao‐Ping Yang:** performed echocardiography; **Jiang Xu:** addressed reviewer's critiques; **Mariela Mendez:** analyzed data, provided manuscript comments, addressed reviewer's critiques; **Pablo A. Ortiz:** supervision, acquired funding, edited manuscript.

## Funding

This work was supported by the National Institute of Diabetes and Digestive and Kidney Diseases, R01DK131114A1. National Heart, Lung, and Blood Institute, NIH R01HL136456‐A1. National Institute on Aging, NIH K99AG075161. American Heart Association, 16PRE27510032.

## Conflicts of Interest

The authors declare no conflicts of interest.

## Data Availability

The data that support the findings of this study are available from the corresponding author upon reasonable request.
